# Importancia pronóstica de las mutaciones del gen promotor de la transcriptasa inversa de la telomerasa en los meningiomas de alto grado

**DOI:** 10.7705/biomedica.6100

**Published:** 2022-12-01

**Authors:** Alejandro Cañas, Enrique Jiménez, Fernando Hakim, Juan Armando Mejía, Juan Fernando Ramón, Diego Gómez, Daniel Jaramillo-Velásquez, Sonia Bermúdez, Nicolás Useche, Diego Pineda, Hernando Cifuentes, Antonio Becerra, Álvaro Muñoz, Nicolás Santoyo, Alejandro Ruiz-Patiño, Carolina Sotelo, Pilar Archila, July Rodríguez, Jenny Ávila, Camila Ordóñez-Reyes, Juan Esteban García-Robledo, Luisa Ricaurte, Leonardo Rojas, Óscar Feo, Remberto Burgos, Carlos Ramírez, Óscar Arrieta, Lucía Zataraín-Barrón, Carlos Vargas, Hernán Carranza, Jorge Otero, Andrés F. Cardona

**Affiliations:** 1 Departamento de Neurocirugía, Fundación Santa Fe de Bogotá, Bogotá, D.C., Colombia Departamento de Neurocirugía Fundación Santa Fe de Bogotá Bogotá, D.C. Colombia; 2 Departamento de Imágenes Diagnósticas, Sección Neuroradiología, Fundación Santa Fe de Bogotá, Bogotá, D.C., Colombia Departamento de Imágenes Diagnósticas Sección Neuroradiología Fundación Santa Fe de Bogotá Bogotá, D.C. Colombia; 3 Departamento de Radiología, Sección Neuroradiología, Clínica del Country, Bogotá, D.C., Colombia Departamento de Radiología Sección Neuroradiología Clínica del Country Bogotá, D.C. Colombia; 4 Departamento de Neurocirugía, Clínica del Country, Bogotá, D.C., Colombia Departamento de Neurocirugía Clínica del Country Bogotá, D.C. Colombia; 5 Departamento de Neurocirugía, Clínica Colsanitas, Bogotá, D.C., Colombia Departamento de Neurocirugía Clínica Colsanitas Bogotá, D.C. Colombia; 6 Departamento de Radiooncología, Instituto de Cáncer Carlos Ardila Lülle, Fundación Santa Fe de Bogotá, Bogotá, D.C., Colombia Departamento de Radiooncología Instituto de Cáncer Carlos Ardila Lülle Fundación Santa Fe de Bogotá Bogotá, D.C.; 7 Fundación para la Investigación Clínica y Molecular Aplicada del Cáncer - FICMAC, Bogotá, D.C., Colombia Fundación para la Investigación Clínica y Molecular Aplicada del Cáncer - FICMAC Bogotá, D.C. Colombia; 8 Grupo de Investigación en Oncología Molecular y Sistemas Biológicos, Universidad El Bosque, Bogotá, D.C., Colombia Universidad El Bosque Grupo de Investigación en Oncología Molecular y Sistemas Biológicos Universidad El Bosque Bogotá, D.C. Colombia; 9 División de Hematología y Oncología, Clinica Mayo, Scottsdale, AZ, Estados Unidos División de Hematología y Oncología Clinica Mayo Scottsdale AZ Estados Unidos; 10 Departamento de Patología, Clínica Mayo, Rochester, MN, Estados Unidos Departamento de Patología Clínica Mayo Rochester MN Estados Unidos; 11 Departamento de Oncología Clínica, Clínica Colsanitas, Bogotá, D.C., Colombia Departamento de Oncología Clínica Clínica Colsanitas Bogotá, D.C. Colombia; 12 Grupo Oncología Clínica y Traslacional, Clínica del Country, Bogotá, D.C., Colombia Grupo Oncología Clínica y Traslacional Clínica del Country Bogotá, D.C. Colombia; 13 Laboratorio Oncología Personalizada, Instituto Nacional de Cancerología, Ciudad de México, México Laboratorio Oncología Personalizada Instituto Nacional de Cancerología Ciudad de México México

**Keywords:** meningoma, mutación con ganancia de función, telomerasa, Meningioma, gain-of-function mutation, telomerase

## Abstract

**Objetivo.:**

Estimar la importancia pronóstica de las mutaciones de la transcriptasa inversa de la telomerasa en pacientes colombianos con meningiomas de grados II y III.

**Materiales y métodos.:**

Es un estudio de cohorte, retrospectivo y multicéntrico, que incluyó pacientes con diagnóstico de meningioma persistente o recidivante, de grados II y III, según la clasificación de la OMS, reclutados entre el 2011 y el 2018, con tratamiento sistémico (sunitinib, everolimus con octreótido o sin él, y bevacizumab). El estado de la mutación del promotor de la transcriptasa inversa de la telomerasa se determinó por medio de la PCR.

**Resultados.:**

Se incluyeron 40 pacientes, en 21 (52,5 %) de los cuales se encontraron mutaciones en la transcriptasa inversa de la telomerasa, siendo las variantes más frecuentes la C228T (87,5 %) y la C250T (14,3 %). Estas fueron más frecuentes entre los pacientes con meningiomas anaplásicos (p=0,18), en aquellos con más de dos recurrencias (p=0,04), y en los que presentaron lesiones en la región parasagital y la fosa anterior (p=0,05). Los sujetos caracterizados por tener alteraciones puntuales fueron tratados con mayor frecuencia con la serie de medicamentos everolimus, sunitinib y bevacizumab (p=0,06). Tras el inicio del tratamiento médico, la supervivencia global fue de 23,7 meses (IC_95%_ 13,1-34,2) en los pacientes con mutaciones y, de 43,4 meses (IC_95%_ 37,5-49,3), entre aquellos sin mutaciones (p=0,0001). Los resultados del análisis multivariado demostraron que, únicamente, el número de recurrencias y la presencia de mutaciones en el gen de la transcriptasa inversa de la telomerasa, fueron factores que afectaron negativamente la supervivencia global.

**Conclusiones.:**

Las mutaciones en el gen promotor de la transcriptasa inversa de la telomerasa permiten identificar los pacientes con alto riesgo, cuya detección podría ser de utilidad para seleccionar el mejor esquema terapéutico.

## Abstract (en)

**Introduction:**

Mutations in the promoter region of telomerase reverse transcriptase occur frequently in meningiomas.

**Objective:**

To estimate the prognostic importance of telomerase reverse transcriptase mutations in Colombian patients with grades II and III meningioma.

**Materials and methods:**

This was a multicenter retrospective cohort study of patients diagnosed with refractory or recurrent WHO grades II and III meningiomas, recruited between 2011 and 2018, and treated with systemic therapy (sunitinib, everolimus ± octreotide, and bevacizumab). Mutation status of the telomerase reverse transcriptase promoter was established by PCR.

**Results:**

Forty patients were included, of which telomerase reverse transcriptase mutations were found in 21 (52.5%), being C228T and C250T the most frequent variants with 87.5 % and 14.3 %, respectively. These were more frequent among patients with anaplastic meningiomas (p=0.18), with more than 2 recurrences (p=0.04); and in patients with parasagittal region and anterior fossa lesions (p=0.05). Subjects characterized as having punctual mutations were more frequently administered with everolimus, sunitinib and bevacizumab drug series (p=0.06). Overall survival was 23.7 months (CI_95%_ 13.1-34.2) and 43.4 months (CI_95%_ 37.5-49.3; p=0.0001) between subjects with and without mutations, respectively. Multivariate analysis showed that the number of recurrences and the presence of telomerase reverse transcriptase mutations were the only variables that negatively affected overall survival.

**Conclusions:**

Mutations in telomerase reverse transcriptase allows the identification of high-risk patients and could be useful in the selection of the best medical treatment.

Los meningiomas representan ~40 % de los tumores intracraneales, con una incidencia global próxima a los 8,1 casos por 100.000 habitantes (*Central Brain Tumor Registry of the United States*, CBTRUS). La mayoría son lesiones de bajo grado [grado I, según la clasificación de la Organización Mundial de la Salud, (OMS)]; no obstante, una pequeña fracción de las neoplasias meningoteliales se categorizan como lesiones de alto grado, particularmente, tumores atípicos (OMS II, 5-20 %) y anaplásicos (OMS III, 1-3 %) ^1^^,^^2^. En los Estados Unidos, se diagnostican anualmente 370 adultos con meningiomas de alto grado, 2.690 sufren actualmente de la enfermedad y la supervivencia global a 5 años se estima en el 63 % ^3^.

Los meningiomas de grado II incluyen variantes morfológicas atípicas, con células claras y coroideas, definidos clásicamente por un índice mitótico de 4 a 19 mitosis por 10 campos de mayor aumento, por la invasión cerebral o por la presencia de tres a cinco características histopatológicas asociadas con atipia ^2^^,^^4^. Estas incluyen crecimiento laminar, necrosis, alteración en la relación entre núcleo y citoplasma, nucléolos prominentes y mayor celularidad ^2^.

Usualmente, la presencia de 1 o 2 características atípicas en un meningioma de grado I, se asocia con un mayor riesgo de progresión y recurrencia ^(^^5^. Las neoplasias anaplásicas tienen un índice mitótico de 20 o más por 10 campos de mayor aumento, e incluyen las variantes papilar y rabdoide ^5^^,^^6^.

Con el advenimiento de la genómica tumoral, se han realizado esfuerzos para mitigar la recurrencia de los meningiomas de alto grado, incluyendo la identificación de blancos moleculares potencialmente modulables. Inicialmente, se identificaron numerosas mutaciones puntuales, deleciones y reordenamientos asociados con la neurofibromina (*NF*_*2*_ en 22q12.2) ^7^, lo que explica cerca del 50 % de los casos esporádicos. Además, se han documentado alteraciones recurrentes en *SMO*, *AKT*_*1*_ (*E17K*), *PIK*_*3*_ *CA*, *POLR*_*2*_ *A*, *SMARCB*_*1*_ , *SWI-SNF*, *KLF*_*4*_ y *TRAF*_*7*_ (*K409Q*) ^8^^-^^10^. Estas alteraciones son típicas de los tumores de bajo grado, pero suelen estar ausentes en los meningiomas atípicos y anaplásicos ^11^.

Por el contrario, los meningiomas de alto grado presentan ganancias y pérdidas cromosómicas, y alteraciones en el número de copias. En particular, suelen tener pérdidas en el cromosoma 22 o modificaciones alélicas en 1p, 6q, 10, 14q y 18q. La pérdida de 1p predice un comportamiento biológico más agresivo, excepto en los tumores anaplásicos de patrón rabdoide. De forma similar, las pérdidas en 9p21, donde se codifican los inhibidores dependientes de ciclinas *CDKN*_*2*_ *A* y *CDKN*_*2*_ *B*, sugieren una mayor probabilidad de cambio en el grado de los tumores de grado I y II ^12^^,^^13^. En la figura suplementaria 1, se muestra el espectro de alteraciones cromosómicas de los meningiomas.

La disrupción del genoma exhibe una fuerte asociación con la ubicación del tumor primario, independientemente del grado tumoral. Los meningiomas de la base del cráneo no suelen mostrar alteraciones en el número de copias, mientras que, en los de la convexidad (falcina y parasagital), se presentan abundantes alteraciones cromosómicas; los de la región lateral se encuentran en un punto intermedio. Estos patrones sugieren que las mutaciones de los meningiomas de alto grado se agrupan en diversas ubicaciones anatómicas, lo que sugiere ontogenias divergentes ^14^. En concordancia, los meningiomas de alto grado se asocian con pocas mutaciones somáticas recurrentes, incluyendo *NF*_*2*_ , *SMARCE*_*1*_ , *BAP*_*1*_ y el gen de la transcriptasa inversa de la telomerasa (*TERT*) ^15^; es más frecuente en las lesiones secundarias y tiene una expresión diferencial en diferentes regiones geográficas ^14^^-^^18^.

La pérdida de la capacidad de mantener los telómeros se considera una característica distintiva de las neoplasias, dado que, en más del 90 % de las células malignas, se sobreexpresa la enzima telomerasa, la cual contrarresta activamente su acortamiento ^19^. Las mutaciones del gen promotor de la transcriptasa inversa de la telomerasa (*TERTp*, 5p15.33), se descubrieron inicialmente en los melanomas y, posteriormente, se documentaron en diversos tumores sólidos, entre ellos, los meningiomas de alto grado. Además de las mutaciones en *TERTp*, la expresión aberrante de la telomerasa puede ser causada por reordenamientos del gen por amplificación, fusiones o metilación ^19^.

Las alteraciones en el *TERT* se traducen en un aumento significativo de la expresión funcional de la proteína que tiene cuatro dominios: el N-terminal, esencial para la telomerasa, el dominio de la transcriptasa inversa, el de unión al ARN y el de la extensión C-terminal ^14^. Las alteraciones más comunes son las mutaciones puntuales C250T y C228T, localizadas en la región promotora del gen ^19^. Un metaanálisis que incluyó los datos individuales de 677 pacientes con meningiomas de grados I a III, proporcionó evidencia de que las mutaciones en *TERTp* constituyen un factor pronóstico negativo, independientemente del grado tumoral ^20^. Los estudios iniciales para valorar la correlación genómica, los cuales incluyeron entre 21 y 58 pacientes, demostraron que la frecuencia de mutaciones en *TERTp* oscila entre el 14 y el 23 % ^20^^-^^24^. Por el momento, solo en un estudio se comparó la expresión de mutaciones en *TERTp* en un subgrupo de pacientes con meningiomas de alto grado, primarios y secundarios, y se sugirió que dichas mutaciones promueven la evolución clonal de las neoplasias meningoteliales ^23^.

Las dos mutaciones encontradas en *TERTp* permiten mantener la longitud del telómero, inmortalizando la célula tumoral mediante la formación de una superfamilia de factores de transcripción conocida como E26/complejo del factor ternario (ETS/TFC), evento que facilita la sobrerregulación del gen. Otro suceso que facilita el aumento de la expresión del ARNm de *TERT* es la hipermetilación del promotor ^20^.

En pocos estudios se ha validado la importancia pronóstica de la transcriptasa inversa de la telomerasa en pacientes con meningiomas de alto grado, tratados con diversas intervenciones farmacológicas en serie. De igual manera, se desconoce la frecuencia de las mutaciones puntuales C228T y C250T en meningiomas de alto grado originados en poblaciones hispanas de múltiple ancestro.

El presente estudio incluye el análisis detallado de 40 pacientes con meningiomas de alto grado caracterizados según la transcriptasa inversa de la telomerasa, tratados y seguidos de forma homogénea, en dos instituciones de Bogotá, Colombia.

## Materiales y métodos

### Diseño y población de estudio

Este es un estudio retrospectivo multicéntrico de cohorte de 40 pacientes seleccionados de un grupo inicial de 59 con diagnóstico de meningioma de grados II y III, confirmado mediante histopatología, y tratados en dos centros de referencia de Bogotá, Colombia, entre diciembre de 2011 y enero de 2018.

Los criterios de inclusión fueron: personas mayores de 18 años y con diagnóstico histológico de meningioma atípico o anaplásico, cuyo tejido estuviese disponible para evaluar el estado de la mutación del gen promotor *TERT*. Los pacientes fueron categorizados en dos grupos para el análisis: *TERT* silvestre y *TERT* mutado. Los registros médicos se revisaron para obtener la información clínica y demográfica.

De los 59 pacientes iniciales, se incluyeron 40 (68 %) con meningiomas agresivos, recurrentes o en progresión, que fueron tratados con: 30 mg intramusculares de acetato de octreótido LAR [O] cada 28 días y 10 mg diarios de everolimus [E] por vía oral, 50 mg de sunitinib [Su] por vía oral durante los días 1 a 28 en un ciclo de 42 días, o 10 mg/kg de bevacizumab [Bev] intravenosos los días 1 y 15. De estos 49 pacientes, los 16 (40 %) expuestos al análogo de somatostatina presentaban sobreexpresión de SSTR2 (receptor 2 de la somatostatina).

La información clínica fue revisada para establecer las características sociodemográficas (edad al momento del diagnóstico, número de recurrencias y compromiso neurológico basal) y las del tratamiento previo (extensión de la resección, tipo y dosis de radioterapia), y los eventos adversos y resultados clínicos.

Todos los procedimientos que hicieron parte del estudio se practicaron bajo los estándares éticos del Comité de Investigación Institucional de la Clínica del Country (LR23-2016), conforme a la declaración de Helsinki de 1964. En todos los casos, se obtuvo la firma previa del consentimiento informado, con el fin de iniciar la recolección de la información clínica, la revisión histopatológica y la caracterización genómica. La tipificación histológica se hizo de acuerdo con los criterios de clasificación para tumores del sistema nervioso central propuestos por la Organización Mundial de la Salud, OMS 2016 (versión 1.0, 2016) ^25^.

En la figura suplementaria 2, se presentan las características histológicas del meningioma atípico y del anaplásico.

En este estudio se incluyeron 40 pacientes mayores de 18 años con evidencia radiológica de progresión tumoral, luego de resección quirúrgica y radioterapia (radioterapia de intensidad modulada o radiocirugía). El tratamiento fue homogéneo, de acuerdo con las recomendaciones actuales con el inhibidor mTOR, solo o en combinación, el inhibidor de tirosina-cinasa multidiana, y el anticuerpo monoclonal antiangiogénico (anti-VEGF). Los tres esquemas terapéuticos estándar, según su secuencia, fueron los siguientes: i) everolimus, sunitinib y bevacizumab (en su orden, de primera, segunda o tercera línea); ii) sunitinib, everolimus y bevacizumab, y iii) everolimus seguido por sunitinib, elegidos según el criterio del médico tratante. El tratamiento se interrumpió por progresión de la enfermedad o por toxicidad dependiente de la dosis, de acuerdo con las recomendaciones del *Common Terminology Criteria for Adverse Events,* versión 4.0 (CTCAE) (https://evs.nci.nih.gov/ftp1/ CTCAE/About.html).

En general, los pacientes fueron evaluados cada tres semanas mediante un examen físico y, cada 8 a 10 semanas, con una resonancia magnética con contraste. La evaluación de la respuesta al tratamiento se basó en los criterios de evaluación radiológica en neurooncología, diseñados para pacientes con meningiomas, que son incluidos en experimentos clínicos ^26^. Durante el seguimiento, se practicaron exámenes de laboratorio de rutina mensualmente o en menos tiempo, según el criterio médico.

### Aislamiento del ADN

EL ADN genómico se extrajo a partir de tejido tumoral embebido en parafina microseccionado (3 mm) y fijado con formalina utilizando el Genomic DNA Purification Kit (Thermo Scientific™, Catalog number K0512). El equipo de patología tumoral analizó las muestras para garantizar un contenido de células tumorales mayor del 90 % y, así, demarcar el área del tumor. El ADN se purificó utilizando el PureLink™ Pro 96 Genomic DNA Purification Kit (Thermo Scientific™, Catalog number K182104A) siguiendo las instrucciones del fabricante y protocolos previamente estandarizados. Posteriormente, el ADN se cuantificó mediante nanoespectrofotometría en un NanoDrop™ ND-2000 (Thermo Scientific™, Waltham, MA) usando una relación de absorbancia de 260/280 nm.

### Análisis de mutaciones en el promotor de TERT

La región promotora de la transcriptasa inversa de la telomerasa (*TERT*) que contiene las mutaciones C228T, C250T, 7, 8 y C229A9, así como el polimorfismo rs2853669 (c.-245T> C), se amplificaron usando 25 ng de ADN genómico previamente extraído con el kit HotStar Taq Master Mix™ y la solución aditiva Q (Qiagen, Alemania), y los cebadores S 5ʹ-AGTGGATTCGCGGGCACAGA-3' y AS 5'-CAGCGCTGCCTGAAACTC-3'.

La amplificación de la PCR dio como resultado un producto de 235 pb. La calidad de los productos se corroboró mediante una electroforesis en gel de poliacrilamida, seguida de una limpieza por PCR, utilizando el kit de un paso Illustra ExoProStar™ (GE Healthcare Life Sciences).

Los productos de la PCR fueron secuenciados usando el kit de secuenciación de ciclos BigDye™ Terminator, versión 1.1, en el analizador genético Applied Biosystems™ 3130 (Applied Biosystems, USA), siguiendo los procedimientos estándar descritos por el productor. Todas las muestras se verificaron en dirección directa e inversa, y se utilizó el *software* SeqScape, versión 3.0 (Applied Biosystems, USA) para el análisis de mutaciones y el ensamblaje de los fragmentos ^27^.

En la figura suplementaria 3 se muestra el esquema de la región promotora de *TERT* con la numeración de los nucleótidos en el cromosoma 5 y, además, la secuencia de ADN de la región *hotspot* de una hebra de tipo salvaje y una mutada, así como los cromatogramas que evidencian las mutaciones heterocigotas C228T y C250T.

### Análisis estadístico

Los resultados se describieron por medio de la determinación de frecuencias absolutas, relativas, medidas de tendencia central y de dispersión. Las variables se analizaron mediante tablas de contingencia sometidas a pruebas de dependencia y asociación, usando la prueba de ji al cuadrado (*X* ^*2*^ ) o el test exacto de Fisher, cuando fue necesario. Para todos los casos, el nivel de significancia estadística se determinó en p<0,05.

Las estimaciones de supervivencia se hicieron utilizando el modelo no paramétrico del límite del producto (método de Kaplan-Meier), y sus funciones se compararon mediante la prueba *log-rank*.

Con el fin de valorar los factores que influyeron sobre la mortalidad, se hizo un análisis multivariado usando el modelo de riesgo proporcional (regresión de Cox). Todos Los análisis estadísticos se hicieron utilizando el programa SPSS™, versión 23.0 (IBM Corp. IBM SPSS Statistics for Windows, Armonk, NY, IBM Corp Released 2015).

## Resultados

### Características demográficas de los pacientes

De los 59 pacientes iniciales, se seleccionaron 40 pacientes con meningiomas atípicos o anaplásicos recurrentes y se incluyeron en el estudio. Sus características demográficas se resumen en el cuadro 1. La mayoría de fueron mujeres, con una distribución de 2 a 1, y hubo predominio del diagnóstico en pacientes mayores de 60 años (52,5 %).

**Cuadro 1:** Características demográficas de los pacientes

Variable		Valor	%, rango o IC_95_%
	Edad (media) (años)	51,3 (DE ± 14,9)	28 a 88
	Mujeres [n (%)]	25	62,5 %
**Patología**		**n**	**%**
	OMS II/atípico recurrente [n (%)]	11	27,5
	OMS III/anaplásico [n (%)]	29	72,5
**Número de recurrencias antes de iniciar el tratamiento médico**		**n**	**%**
	1	8	20,0
	2	11	27,5
	3	10	25,0
	4	8	20,0
	5	3	7,5
**Localización primaria**		**n**	**%**
	Convexidad	4	10,0
	Parasagital	10	25,0
	Fosa anterior	18	45,0
	Fosa media	5	12,5
	Fosa posterior	3	7,5
**Multicentricidad**		10	25
**Número de cirugías previas**		**n**	**%**
	1	4	10,0
	2	23	57,5
	3	7	17,5
	4	3	7,5
	5	3	7,5
**Extensión de la resección**		**n**	**%**
	Total	20	50,0
	Subtotal	20	50,0

Además, 10 de los pacientes tuvieron enfermedad multicéntrica, 8 de los cuales tenían un fenotipo anaplásico con especial localización en la fosa media y posterior (p=0,043).

### Tratamiento

Todos los pacientes fueron sometidos a resección quirúrgica; la mediana del número de cirugías parciales o radicales por paciente fue de 2, con un rango de 1 a 5. En el grupo de pacientes con tumores atípicos (n=11), todos menos uno tuvieron resecciones de grado Simpson III, en comparación con el grupo de sujetos con tumores anaplásicos, entre los cuales el 65 % (n=13) tuvieron procedimientos quirúrgicos de grado Simpson I y II (p=0,0089). No se encontraron diferencias estadísticamente significativas entre los dos grupos, según el número de recurrencias (p=0,36). Además, todos los pacientes fueron tratados con radioterapia de intensidad modulada (n=20, 65 %) (IMRT: frecuencia = 27; porcentaje = 67,5; SRS: frecuencia = 13; porcentaje = 32,5), y el tiempo medio transcurrido entre la radioterapia y el comienzo de la primera línea de tratamiento sistémico fue de 22,9 meses (IC_95%_ 1,8-189,0). De manera similar, no hubo diferencias de significancia estadística en la supervivencia libre de progresión entre los pacientes tratados con radioterapia de intensidad modulada o radiocirugía estereotáctica: 23,6 frente a 19,4 meses (p=0,47), a diferencia de la supervivencia global, la cual fue superior en casos de tumores atípicos que en los de los anaplásicos (p=0,013) (figura suplementaria 4).

Al momento de comenzar la terapia médica, el diámetro medio de los tumores era de 42,3 mm (DE ± 12,2), sin diferencias entre los de grados II y III (p=0,85). Antes de recibir sunitinib o el tratamiento basado en everolimus, un sujeto (3,2 %) (1 de 40 es el 2,5 %) recibió hidroxiurea/imatinib, sin ningún beneficio. Diecinueve (61,3 %) (19 de 40 es el 47,5 %) pacientes fueron tratados con everolimus o everolimus más octreótrido como medicamentos de primera línea y 11 (35,5 %) (11 de 40 es el 27,5 %) con sunitinib. La relación para la segunda línea se invirtió: 18 (58,1 %) (18 de 40 es el 47,5 %) pacientes recibieron sunitinib, 4 (12,9 %) (4 de 40 es el 10,0 %), tratamiento basado en everolimus, y 1 (3,2 %) (1 de 40 es el 2,5 %), bevacizumab. Ocho (25, 8 %) (8 de 40 es el 20,0 %) pacientes siguen al momento de realizar el estudio aún con el tratamiento de primera línea. Después de una mediana de tiempo de seguimiento de 31,8 meses (IC_95%_ 13,6-47), 10 pacientes (32,3 %) recibieron los medicamentos en el orden everolimus, sunitinib, bevacizumab; 9 pacientes (29,0 %) fueron tratados con everolimus y posteriormente sunitinib, y 4 (12,9 %) iniciaron con sunitinib seguido de everolimus y finalizaron con bevacizumab.

### Resultados del tratamiento

**Figura 1:**
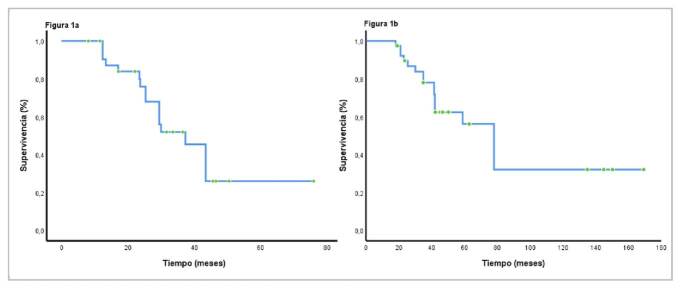
a. Supervivencia global después del inicio del tratamiento médico. b. Supervivencia global después del diagnóstico de la enfermedad

**Figura 2:**
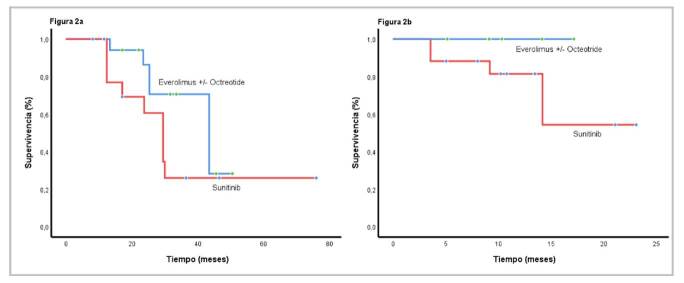
a. Mediana de la supervivencia global según el tratamiento de primera línea: con everolimus (36 meses; IC_95%_ 25,3-41,7) y con sunitinib (29,5 meses; IC_95%_ 22,5-37,5) (p=0,349). b. Mediana de la supervivencia libre de progresión de acuerdo con el tratamiento de primera línea: con everolimus (12,1 meses; IC_95%_ 9,2-21,1); y con sunitinib (9,1 meses; IC_95%_ 6,8-16,8) (p=0,43)

Después de iniciado el tratamiento médico, la mediana de supervivencia global para la cohorte fue de 37,3 meses (IC_95%_ 28,5-42,1) y, después del diagnóstico inicial, fue de 78,1 meses (IC_95%_ 42,5-98,2) (figura 1, a y b). Al comparar a los pacientes con los tratamientos de primera línea más comunes (everolimus con octeótrido o sin él, o sunitinib), la mediana de la supervivencia global fue de 36 meses (IC_95%_ 25,3-41,7) y de 29,5 meses (IC_95%_ 22,5-37,5), respectivamente (p=0,349) (figura 2a). La supervivencia libre de progresión fue de 12,1 meses (IC_95%_ 9,2-21,1), a diferencia de 9,1 meses (IC_95%_ 6,8-16,8), para las mismas intervenciones, respectivamente p=0,43) (figura 2b).

Por otro lado, de acuerdo con los criterios de evaluación radiológica en neurooncología (meningiomas), la tasa de mejoría con la primera línea fue de 6 (43 %) pacientes que alcanzaron una enfermedad estable y 4 (28,6 %) que lograron una respuesta parcial con everolimus con octeótrido o sin él, en contraste con 7 (63,6 %) que alcanzaron enfermedad estable y 4 (36,4 %) con respuesta parcial cuando recibieron sunitinib.

**Figura 3:**
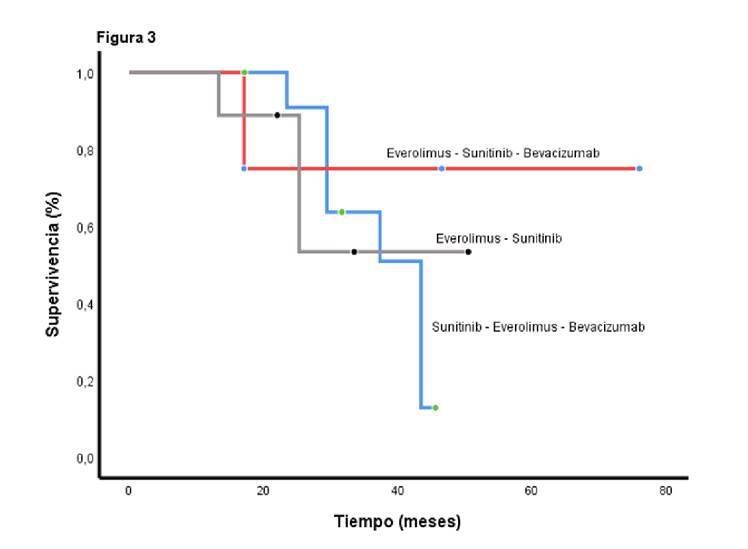
La supervivencia global según la secuencia de tratamiento es de 43,4 meses para la serie everolimus, sunitinib y bevacizumab (IC_95%_ 37,1-49,7), de 37,4 meses para sunitinib, everolimus y bevacizumab (IC_95%_ 26,3-48,0), y de 27,7 meses para sunitinib, bevacizumab y everolimus (IC_95%_ 12,4-46,0) (p=0,0001).

El beneficio clínico (respuesta completa, respuesta parcial o enfermedad estable) mostró una tendencia a prolongar la supervivencia global sin una asociación estadísticamente significativa (p=0,246). La supervivencia libre de progresión para los pacientes que recibieron sunitinib y everolimus con octeótrido o sin él en la segunda línea, fue de 9,13 (IC_95%_ 2,4-13,6) y 10,17 (IC_95%_ 6,13-14,5), respectivamente. La supervivencia global fue superior cuando se administró el orden sunitinib, everolimus y bevacizumab, en contraste con todas las demás posibilidades (p=0,0001) (figura 3), independientemente de la extensión de la cirugía inicial (p=0,94), del tiempo entre el diagnóstico y el inicio del tratamiento médico (menor o mayor de 20 meses) (p=0,17); del número de recurrencias (menos o más de 3 recurrencias) (p=0,47); de la edad (menor o mayor de 65 años) (p=0,64), y del sexo (p=0,30).

**Figura 4:**
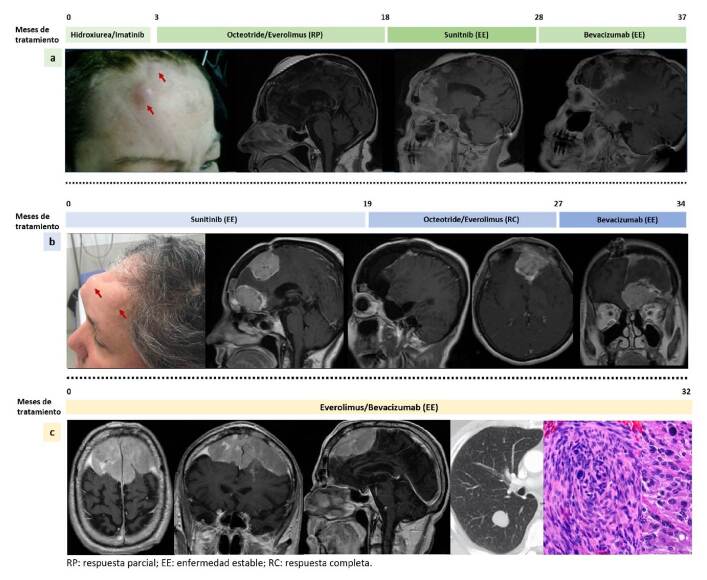
a. Mujer de 54 años con diagnóstico de meningioma anaplásico recurrente en tres oportunidades y con *TERT* silvestre, tratada en tres oportunidades con cirugía, radioterapia de intensidad modulada, radiocirugía, y con la serie de medicamentos everolimus, sunitinib y bevacizumab; con este tratamiento, alcanzó una supervivencia global de 37 meses. b. Mujer de 59 años a quien se le diagnosticó meningioma anaplásico con la mutación *TERT* ^*C228T*^ . Su evolución fue rápida a pesar de dos intervenciones neuroquirúrgicas parciales, de radioterapia de intensidad modulada y de la administración de la serie de medicamentos sunitinib, everolimus y bevacizumab; con este tratamiento se logró una supervivencia global de 39,5 meses, 34 de los cuales estuvo en tratamiento médico. c. Hombre de 47 años con diagnóstico de meningioma anaplásico bifrontal de gran tamaño, con extensión ósea y pulmonar, que presentó la mutación *TERT* ^*C228T*^ . Fue tratado con exéresis parcial, radioterapia de intensidad modulada y la combinación de everolimus y bevacizumab; con este tratamiento, ha tenido una supervivencia global de la enfermedad de 32 meses hasta el momento.

En la figura 4, se muestran la evolución clínica y las imágenes diagnósticas de varios pacientes tratados en el orden everolimus, sunitinib y bevacizumab; sunitinib, everolimus y bevacizumab; y everolimus y bevacizumab.

### Resultados de acuerdo con la presencia de mutaciones en TERT

Se encontraron mutaciones en *TERT* en 21 (52,5 %) pacientes, 18 (85,7 %) con la variante C228T y 3 (14,3 %) con la C250T. Las mutaciones en *TERT* fueron más frecuentes entre los pacientes con meningiomas anaplásicos (17 meningiomas de grado III *versus* 4 tumores de grado II) (p=0,18), en aquellos con más de dos recurrencias (p=0,04), y en los que presentaron lesiones localizadas en la región parasagital y en la fosa anterior (p=0,05). De igual forma, a los sujetos caracterizados por tener alteraciones puntuales en *TERT*, se les administró con mayor frecuencia everolimus seguido de sunitinib y, posteriormente, bevacizumab (n=6) (p=0,06).

**Figura 5:**
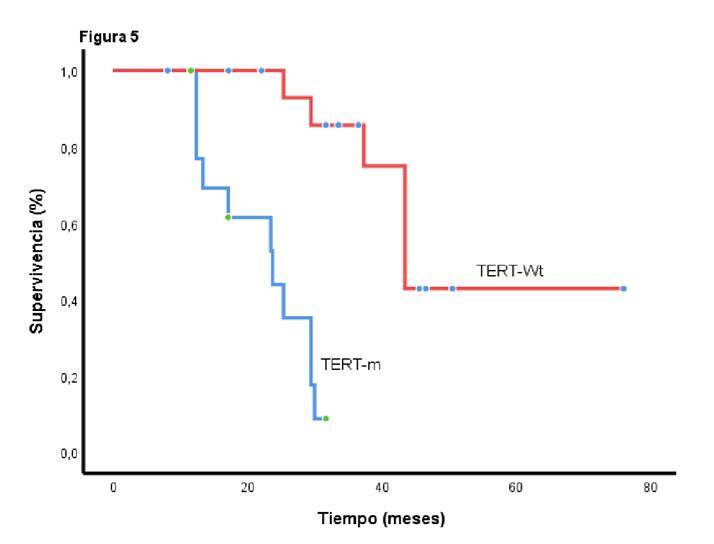
Supervivencia global según la presencia de mutaciones en *TERT*

Tras el inicio del tratamiento médico, la supervivencia global fue de 23,7 meses (IC_95%_ 13,1-34,2) entre los sujetos con mutaciones en *TERT*, y de 43,4 meses (IC_95%_ 37,5-49,3) en aquellos sin estas mutaciones (p=0,0001) (figura 5).

La presencia de estas mutaciones no afectó la supervivencia libre de progresión para la primera (p=0,059), la segunda (p=0,31) y la tercera línea (p=0,09). No obstante, cuando este análisis se hizo exclusivamente para el tratamiento con bevacizumab en segunda y tercera líneas, se encontró que dichas mutaciones influenciaron negativamente la supervivencia global tras la exposición al antiangiogénico (p=0,004). Además, la supervivencia global fue estadísticamente inferior para el subgrupo de pacientes con tumores anaplásicos con mutaciones en *TERT* (p=0,031) (figura suplementaria 5).

En el análisis multivariado, se encontró que solo dos variables impactaron la supervivencia global: el número de recurrencias (OR=10,5; IC_95%_ 1,1-98,3) y la presencia de mutaciones en el *pTERT* (OR=150,9; IC_95%_ 4,7-4756,3). 

## Discusión

Los meningiomas atípicos y anaplásicos se relacionan con una mayor morbimortalidad; la edad, el sexo masculino, el estado funcional (medido por la escala de Karnofsky), el índice mitótico, el número de recurrencias e intervenciones quirúrgicas, y el compromiso del nervio óptico, los principales factores clínicos relacionados con el pronóstico ^28^.

Los meningiomas de alto grado suelen ser más frecuentes en los pacientes jóvenes (la edad media es de 57 años en el momento del diagnóstico) y, usualmente, están relacionados con mutaciones somáticas *de novo* o con la evolución fenotípica y clonal a partir de lesiones de menor grado ^28^.

En general, la localización de la lesión tiende a predecir el grado de diferenciación, teniendo en cuenta que la mayoría de los tumores ubicados en la fosa media y anterior suelen tener un patrón meningotelial típico o transicional. Por el contrario, algunas de las neoplasias de la fosa media y la mayoría de las encontradas en la fosa posterior, presentan mutaciones en el promotor de *TERT* y un linaje secretor.

Globalmente, la frecuencia de alteraciones en *TERT* oscila entre el 14 y el 23 %, siendo mayor en los tumores recurrentes y en las lesiones de alto grado ^23^^,^^24^^,^^29^^,^^30^. La incidencia de los meningiomas anaplásicos catalogados como silvestres y mutados en *TERT*, se ha estimado en 2 y 8 casos por millón de habitantes por año, respectivamente (29).

En el presente estudio se documentó el doble de mutaciones en el *pTERT* (52 %), debido a que la población fue muy seleccionada. Quince de los pacientes que presentaron dichas mutaciones tenían como antecedente dos o más recurrencias y, de ellos, cinco tuvieron más de cuatro eventos quirúrgicos previos.

En concordancia con otras descripciones ^29^^,^^30^, nuestros hallazgos corroboran que la mutación *TERTC228T* fue la alteración más frecuente, y que la multicentricidad fue común en los tumores anaplásicos mutados y recurrentes.

Mirian, *et al*., encontraron una supervivencia libre de recurrencia posterior a la cirugía inicial, de 14 meses para los pacientes con alteraciones puntuales en el *pTERT* y de 101 meses en aquellos sin las mismas ^30^. Además, la razón de riesgo (HR) para recaídas entre los sujetos con mutaciones en *TERT* fue de 3,74 en referencia al grupo con meningiomas silvestres, el cual también presentó una mejoría significativa en la mediana para la supervivencia global (58 meses para los pacientes con mutaciones en el *pTERT versus* 160 meses para los que presentaron la versión silvestre; HR 2,77) (p=0,0001).

El pronóstico de las mutaciones de *pTERT* se confirmó a partir del análisis sistemático de casos, que demostró una tasa de recurrencia 4,8 veces mayor en aquellos sujetos con meningiomas de grado OMS I y II mutados, en comparación con los pacientes portadores de tumores anaplásicos silvestres. De igual forma, la tasa de mortalidad fue 2,7 veces mayor en aquellos pacientes con tumores de grado OMS I y II mutados en el *pTERT*, *versus* el subgrupo de anaplásicos no mutados ^30^.

En la presente serie, el 74 % de los pacientes con mutaciones en el *pTERT* tuvieron una recurrencia en los primeros 20 meses de seguimiento tras la cirugía inicial (p=0,035), y la mediana de supervivencia global desde el diagnóstico fue de 41,5 meses (IC_95%_ 28,1-54,8) en los sujetos con tumores mutados, contra 114 meses (IC_95%_ 83,0-146,0) en la población con tumores silvestres (p=0,0001) (figura suplementaria 6).

Las mutaciones en el *pTERT* también afectaron el pronóstico de la enfermedad (supervivencia global) tras el inicio del tratamiento médico, sin afectar la supervivencia libre de progresión; sin embargo, no fue posible encontrar un efecto determinante sobre este parámetro respecto del tipo de mutación en el *pTERT* (variantes C228T y C250T), posiblemente por la limitación del tamaño de la muestra (p=0,58) (figura suplementaria 7).

Como se describió en el presente estudio, el análisis multivariado para la supervivencia global solo se vio afectado por el número de recurrencias y por las mutaciones en el *pTERT*, información similar a la de otros autores ^30^, que podría permitir categorizar los meningiomas de alto grado para determinar si se requiere radioterapia temprana o diversas intervenciones farmacológicas.

Recientemente, Harmancı, *et al*., propusieron que la aparición de mutaciones en el *pTERT* puede ser un paso temprano en la transición de los meningiomas atípicos ^31^. Como prueba de la evolución clonal, Juratli, *et al*. ^21^, demostraron que la expresión de las mutaciones en *pTERT* puede ser heterogénea en un mismo tumor recurrente según su localización, lo que sustenta el modelo de progresión tumoral acelerado que influye sobre los resultados independientemente del grado tumoral.

En consonancia con las alteraciones en *TERT*, el índice de proliferación medido por el Ki_67_, ha permitido discriminar el pronóstico de los meningiomas agresivos, según el análisis de la supervivencia global y de la supervivencia libre de progresión, después de la radioterapia de intensidad modulada o el tratamiento médico ^32^^,^^33^. Nuestros análisis confirman estos hallazgos, al encontrar un impacto negativo del Ki_67_ mayor o igual al 20 % sobre la supervivencia global, a partir del diagnóstico (p=0,012) o del inicio del tratamiento médico (p=0,013) (figuras no incluidas).

Además de las mutaciones en el *pTERT*, otros factores como las fusiones en LPCAT1-TERT, las pérdidas por heterocigocidad del cromosoma 18q, las deleciones en *CDKN*_*2*_ *A/B*, las mutaciones en *NF*_*2*_ , *ARID*_*1*_ *A,* y *BAP*_*1*_ , así como la expresión de *RB1* ^*S780*^ , también se asocian con un pronóstico desfavorable ^15^^,^^34^^-^^37^.

Recientemente, se ha descrito el papel prometedor de diversos perfiles de metilación para clasificar a los pacientes con meningiomas según su riesgo de recurrencia e impacto sobre resultados como la supervivencia libre de progresión y la global. Olar, *et al*., demostraron por medio de una agrupación no supervisada, que la metilación del *locus* 64-CpG afectó los resultados más representativos independientemente del grado tumoral, el índice mitótico, la escala Simpson, el sexo, la ubicación y el número de copias ^38^. Utilizando métodos similares, Sahm, *et al*., identificaron seis clases únicas de metilación en los diferentes grados del meningioma, documentando un perfil específico para el grado I de alto riesgo y otro para los tumores de grado II de buen pronóstico ^39^.

Múltiples estrategias moleculares, con mayor o menor complejidad, permitirán seleccionar a los pacientes según su riesgo para acelerar el uso de terapias dirigidas o decidir el orden en la administración de los tratamientos después de la cirugía y la radioterapia.

Gracias al conocimiento adquirido sobre las características citogenéticas basales de los meningiomas de alto grado, su heterogeneidad y capacidad de escape ¿del sistema inmunológico por medio de variaciones subclonales que explican la sensibilidad y resistencia a los diferentes tratamientos, hoy disponemos de tratamientos diferentes a la cirugía, la radioterapia y la quimioterapia clásica ^40^. Un número significativo de los meningiomas de alto grado presentan pérdidas en 22q, que se asocian con la presentación de mutaciones supresoras en *NF2*, gen que codifica la neurofibromina 2 (merlina), proteína implicada en las vías de señalización intracelular PI3K/ AKT/mTOR y, de forma paralela con eIF3c, CD44, la proteína cinasa A y p21.

De forma similar, hasta el 70 % de los meningiomas muestran sobreexpresión de SSTR2A, lo que sugiere un entorno propicio para el uso de medicamentos como el everolimus y el octeótrido ^41^^,^^42^. Previamente, Graillon *et al*., demostraron *in vitro* que el everolimus disminuía la viabilidad celular de los meningiomas agresivos, induciendo de forma concomitante la activación de AKT, lo que generó un efecto antiproliferativo paradójico. Este evento se corrigió con la inhibición cooperativa entre el everolimus y el octeótrido, análogo que revirtió la fosforilación de AKT, la transducción por medio de 4EB-P1, y el control del ciclo celular por p27Kip1 y la ciclina D1 ^42^.

Con base en estos hallazgos, el mismo grupo diseñó y llevó a cabo el estudio CEVOREM ^43^ que demostró, en 20 pacientes con tumores recurrentes (18 de grado OMS II y III), que el uso de la combinación de everolimus y octeótrido alcanzó una supervivencia libre de progresión a los 6 meses del 55 % (IC_95%_ 31,3-73,5) y una global a los 6 y 12 meses del 90 % (IC_95%_ 65,6-94,7) y del 75 % (IC_95%_ 50-88), respectivamente. Además, la tasa de respuesta global fue del 78 % después de 3 meses de intervención. Anteriormente, nuestro grupo demostró una supervivencia libre de progresión de 12,1 meses (IC_95%_ 9,2-21,1) para el mismo esquema terapéutico, datos similares a los descritos previamente ^44^.

Usualmente, más del 80 % de los meningiomas agresivos presentan sobreexpresión de VEGFR y PDGFR-β, y cerca del 50 %, de Axl y EGFR ^45^. A partir de esta información, se intentó el uso del sunitinib, un inhibidor multidiana de la tirosina cinasa, en 36 pacientes con meningiomas de alto grado (30 atípicos y 6 anaplásicos) e historia de múltiples recurrencias (mediana de 5; rango de 2 a 10) ^46^. La supervivencia libre de progresión a los 6 meses fue del 42 %, la mediana de la supervivencia libre de progresión fue de 5,2 meses (IC_95%_ 2,8-8,3), y la de la supervivencia global fue 24,6 meses (IC_95%_ 16,5-38,4). La expresión de VEGFR2 predijo una supervivencia libre de progresión de 1,4 meses para los casos negativos y de 6,4 para los positivos (p=0,005) ^46^. En el mismo sentido, nosotros encontramos una supervivencia libre de progresión de 9,1 meses (IC_95%_ 6,8-16,8) para el sunitinib utilizado como medicamento de primera línea.

El presente estudio permitió demostrar un beneficio significativo para la supervivencia global según la secuencia del tratamiento, a favor de la serie everolimus, sunitinib y bevacizumab; seguida por sunitinib, everolimus y bevacizumab, y, por último, sunitinib, bevacizumab y everolimus.

Por el momento, la información integrada para el bevacizumab sigue siendo limitada, que ha permitido encontrar una supervivencia libre de progresión de 16,8 meses (IC_95%_ 6,5-22) y una supervivencia libre de progresión a los 6 meses del 73 % (IC_95%_ 44-93) ^47^^,^^48^.

Nuestros datos demuestran que las mutaciones en el *pTERT* no afectan la supervivencia libre de progresión con respecto a la primera, la segunda y la tercera línea. No obstante, dichas mutaciones influenciaron negativamente la supervivencia global tras la exposición al antiangiogénico.

Para todos los meningiomas, el tratamiento de elección sigue siendo la resección quirúrgica óptima, particularmente, en los casos que presentan tumores con un tamaño mayor de 4 cm, en aquellos que tienen una tasa de crecimiento mayor o igual al 20 % en presencia de un diámetro mayor de menos de 2,5 cm, en aquellos pacientes que exhiben signos de invasión ósea o cerebral, y en los que tienen un aumento de tamaño de más de 1 cm en un año ^49^.

Después del procedimiento neuroquirúrgico, la adición de la radioterapia proporciona una supervivencia libre de progresión a 5 años del 76,5 % en casos de meningiomas atípicos y del 56 % en los de los anaplásicos. Además, la supervivencia global a 5 años es del 77 % para los pacientes con tumores de grado OMS II y del 44 % para los de grado III, punto en el que la toxicidad acumulada oscila entre el 12 % y 35 % ^49^.

Por el momento, la radioterapia ha sustituido a la cirugía como primera elección en tumores pequeños de la base del cráneo o en aquellos con afectación de estructuras neurovasculares (nervio óptico, seno cavernoso o ambos). Sin embargo, la administración de diversos medicamentos sigue estando reservada para los pacientes con tumores agresivos, recurrentes y que no mejoran con la cirugía y la radiación.

Estudios como el presente contribuyen a la construcción del conocimiento necesario para optimizar el tratamiento de los meningiomas agresivos, explorando nuevos blancos terapéuticos y caracterizando el comportamiento biológico de la enfermedad según su perfil molecular ^50^^,^^51^.

En conclusión, el presente estudio aporta datos novedosos sobre la caracterización de los meningiomas de alto grado, por primera vez en una población hispana. Además, resalta la importancia del análisis de las mutaciones del *pTERT* al impactar de forma significativa la supervivencia global en aquellos casos con múltiples recurrencias, y podría ser útil para seleccionar la mejor secuencia del tratamiento médico, utilizando fármacos como el everolimus con octeótrido o sin él, el sunitinib y el bevacizumab. Se requieren estudios adicionales que evalúen la importancia pronóstica de otros biomarcadores en las diferentes etapas de la evolución natural del meningioma y, a su vez, determinar factores predictores de respuesta a tratamientos de tipo sistémico y a la radioterapia.

## Figures and Tables

**Figura suplementaria 1.**
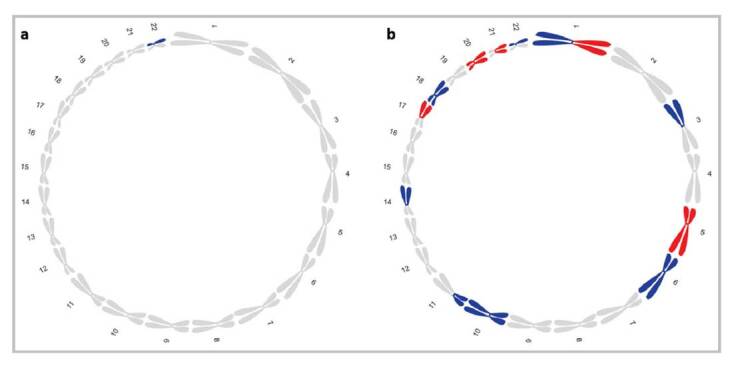
Alteraciones cromosómicas más frecuentes en los meningiomas de grado I-III. **a.** La monosomía 22 es la alteración más común en los meningiomas, relacionada con una ganancia en el número de copias y, con frecuencia, el único evento en los tumores de grado I. **b.** Las alteraciones recurrentes en el número de copias de los cromosomas coloreados son una característica distintiva de los meningiomas de alto grado. El azul denota la pérdida cromosómica y, el rojo, la ganancia.

**Figura suplementaria 2.**
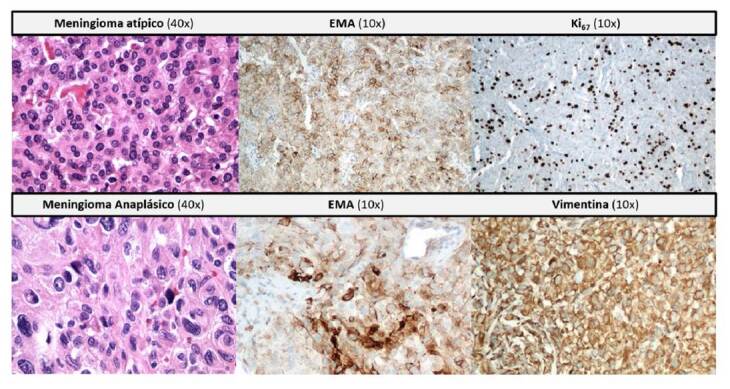
En la franja superior, se muestra la secuencia patológica de un meningioma atípico (OMS II) con un alto índice mitótico (mayor o igual a 4/10 campos de mayor aumento); arquitectura de laminación, formación de células pequeñas, “hipercelularidad”, macronucléolos y necrosis. Las imágenes ilustran, además, la proliferación medida por el Ki67, al igual que la expresión del antígeno epitelial de membrana (EMA). En la porción inferior de la imagen, se muestra la histología de un meningioma anaplásico (OMS III) con más de 20 mitosis por 10 campos de mayor aumento, importante pleomorfismo celular y nuclear, y aspecto sarcomatoide.

**Figura suplementaria 3.**
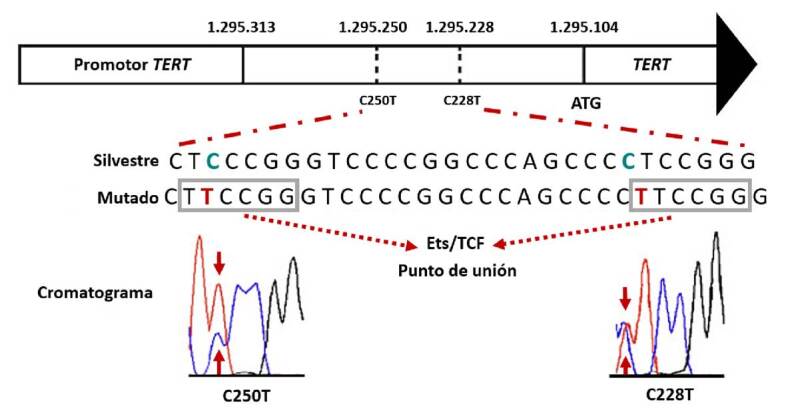
Esquema de la región promotora de *TERT* con la numeración de los nucleótidos en el cromosoma 5. Se muestra la secuencia de ADN de la región *hotspot* con una hebra de tipo silvestre y una mutada, donde se evidencia el intercambio de una citosina por una timina (representada en rojo). Cada mutación conduce a un nuevo punto de unión para los factores de transcripción de E-26 y para los complejos de los factores ternarios (Ets/TCF) (resaltados por rectángulos de color gris). Los cromatogramas de secuenciación muestran las mutaciones heterocigotas C228T y C250T (indicadas por las flechas).

**Figura suplementaria 4.**
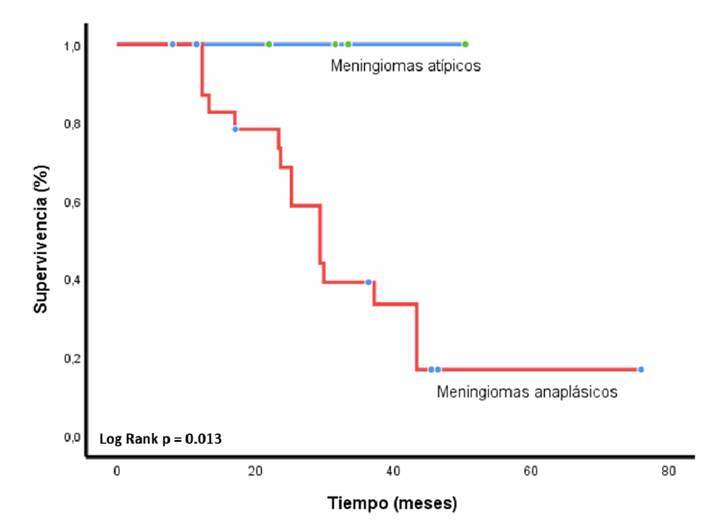
Supervivencia global en casos de meningiomas atípicos y anaplásicos a partir del inicio del tratamiento médico

**Figura suplementaria 5.**
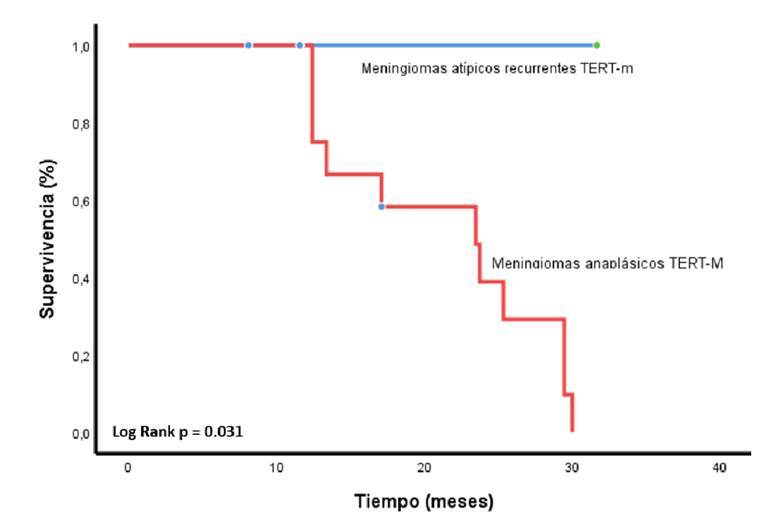
Supervivencia global a partir del inicio del tratamiento según la presencia de mutaciones en el *pTERT* y el grado tumoral (atípicos y anaplásicos)

**Figura suplementaria 6.**
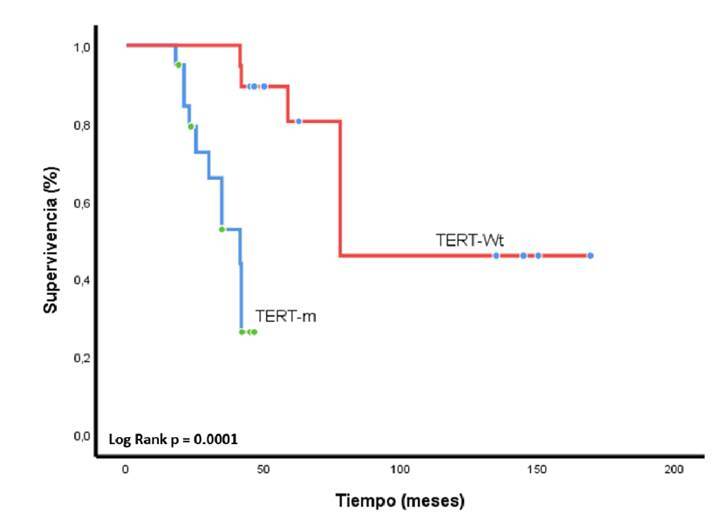
Supervivencia global desde el diagnóstico, según la presencia de mutaciones en el *pTERT*

**Figura suplementaria 7.**
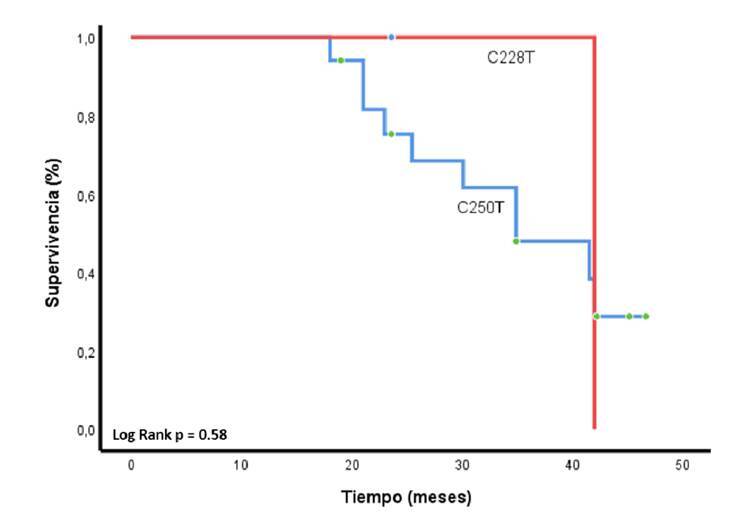
Supervivencia global desde el diagnóstico, de los pacientes con mutaciones en el *pTERT*, según su variante

## References

[1] Perry A, Scheithauer BW, Stafford SL, Lohse CM, Wollan PC. (1999). “Malignancy” in meningiomas: A clinicopathologic study of 116 patients, with grading implications. Cancer.

[2] Louis DN, Ohgaki H, Wiestler OD, Cavenee WK, Burger PC, Jouvet A (2007). The 2007 WHO classification of tumours of the central nervous system. Acta Neuropathol.

[3] Ostrom QT, Gittleman H, Liao P, Vecchione-Koval T, Wolinsky Y, Kruchko C (2017). CBTRUS Statistical Report: Primary brain and other central nervous system tumors diagnosed in the United States in 2010-2014. Neuro Oncol.

[4] Olar A, Wani KM, Sulman EP, Mansouri A, Zadeh G, Wilson CD (2015). Mitotic index is an independent predictor of recurrence-free survival in meningioma. Brain Pathol.

[5] Wang YC, Chuang CC, Wei KC, Chang CN, Lee ST, Wu CT (2016). Long term surgical outcome and prognostic factors of atypical and malignant meningiomas. Sci Rep.

[6] Durand A, Labrousse F, Jouvet A, Bauchet L, Kalamaridès M, Menei P (2009). WHO grade II and III meningiomas: A study of prognostic factors. J Neurooncol.

[7] Agnihotri S, Suppiah S, Tonge PD, Jalali S, Danesh A, Bruce JP (2017). Therapeutic radiation for childhood cancer drives structural aberrations of NF2 in meningiomas. Nat Commun.

[8] Christiaans I, Kenter SB, Brink HC, van Os TA, Baas F, van den Munckhof P (2011). Germline SMARCB1 mutation and somatic NF2 mutations in familial multiple meningiomas. J Med Genet.

[9] Bi WL, Abedalthagafi M, Horowitz P, Agarwalla PK, Mei Y, Aizer AA (2016). Genomic landscape of intracranial meningiomas. J Neurosurg.

[10] Clark VE, Erson-Omay EZ, Serin A, Yin J, Cotney J, Ozduman K (2013). Genomic analysis of non-NF2 meningiomas reveals mutations in TRAF7, KLF4, AKT1, and SMO. Science.

[11] Yuzawa S, Nishihara H, Tanaka S. (2016). Genetic landscape of meningioma. Brain Tumor Pathol.

[12] Bi WL, Greenwald NF, Abedalthagafi M, Wala J, Gibson WJ, Agarwalla PK (2017). Erratum: Genomic landscape of high-grade meningiomas. NPJ Genom Med.

[13] Perry A, Banerjee R, Lohse CM, Kleinschmidt-DeMasters BK, Scheithauer BW. (2002). A role for chromosome 9p21 deletions in the malignant progression of meningiomas and the prognosis of anaplastic meningiomas. Brain Pathol.

[14] Youngblood MW, Miyagishima DF, Jin L, Gupte T, Li C, Duran D (2021). Associations of meningioma molecular subgroup and tumor recurrence. Neuro Oncol.

[15] Shankar GM, Abedalthagafi M, Vaubel RA, Merrill PH, Nayyar N, Gill CM (2017). Germline and somatic BAP1 mutations in high-grade rhabdoid meningiomas. Neuro Oncol.

[16] Smith MJ, O’Sullivan J, Bhaskar SS, Hadfield KD, Poke G, Caird J (2013). Loss-of-function mutations in SMARCE1 cause an inherited disorder of multiple spinal meningiomas. Nat Genet.

[17] Tauziede-Espariat A, Parfait B, Besnard A, Lacombe J, Pallud J, Tazi S (2018). Loss of SMARCE1 expression is a specific diagnostic marker of clear cell meningioma: A comprehensive immunophenotypical and molecular analysis. Brain Pathol.

[18] Vasudevan H, Braunstein S, Phillips JJ, Pekmezci M, Wu A, Reis G (2017). GENE-04. Comprehensive genomic characterization of aggressive meningiomas identifies molecular signatures that predict clinical outcomes. Neuro-Oncology.

[19] Barthel FP, Wei W, Tang M, Martínez-Ledesma E, Hu X, Amin SB (2017). Systematic analysis of telomere length and somatic alterations in 31 cancer types. Nat Genet.

[20] Yuan P, Cao J lin, Abuduwufuer A, Wang LM, Yuan XS, Lv W (2016). Clinical characteristics and prognostic significance of TERT promoter mutations in cancer: A cohort study and a meta-analysis. PLoS ONE.

[21] Juratli TA, Thiede C, Koerner MVA, Tummala SS, Daubner D, Shankar GM (2017). Intratumoral heterogeneity and TERT promoter mutations in progressive/higher-grade meningiomas. Oncotarget.

[22] Peyre M, Gauchotte G, Giry M, Froehlich S, Pallud J, Graillon T (2018). De novo and secondary anaplastic meningiomas: A study of clinical and histomolecular prognostic factors. Neuro Oncol.

[23] Sahm F, Schrimpf D, Olar A, Koelsche C, Reuss D, Bissel J (2016). TERT promoter mutations and risk of recurrence in meningioma. J Natl Cancer Inst.

[24] Spiegl-Kreinecker S, Lötsch D, Neumayer K, Kastler L, Gojo J, Pirker C (2018). TERT promoter mutations are associated with poor prognosis and cell immortalization in meningioma. Neuro Oncol.

[25] Louis DN, Perry A, Reifenberger G, von Deimling A, Figarella-Branger D, Cavenee WK (2016). The 2016 World Health Organization Classification of Tumors of the Central Nervous System: A summary. Acta Neuropathol.

[26] Huang RY, Bi WL, Weller M, Kaley T, Blakeley J, Dunn I (2019). Proposed response assessment and endpoints for meningioma clinical trials: Report from the Response Assessment in Neuro-Oncology Working Group. Neuro Oncol.

[27] Killela PJ, Reitman ZJ, Jiao Y, Bettegowda C, Agrawal N, Diaz LA (2013). TERT promoter mutations occur frequently in gliomas and a subset of tumors derived from cells with low rates of self-renewal. Proc Natl Acad Sci USA.

[28] Apra C, Peyre M, Kalamarides M. (2018). urrent treatment options for meningioma. Expert Rev Neurother.

[29] Goutagny S, Nault JC, Mallet M, Henin D, Rossi JZ, Kalamarides M. (2014). High incidence of activating TERT promoter mutations in meningiomas undergoing malignant progression. Brain Pathol.

[30] Mirian C, Duun-Henriksen AK, Juratli T, Sahm F, Spiegl-Kreinecker S, Peyre M (2020). Poor prognosis associated with TERT gene alterations in meningioma is independent of the WHO classification: An individual patient data meta-analysis. J Neurol Neurosurg Psychiatry.

[31] Harmancı AS, Youngblood MW, Clark VE, Coşkun S, Henegariu O, Duran D (2018). Integrated genomic analyses of de novo pathways underlying atypical meningiomas. Nat Commun.

[32] Maier A, Brøchner CB, Bartek J, Eriksson F, Ugleholdt H, Broholm H (2020). Mitotic and proliferative indices in WHO grade III meningioma. Cancers (Basel).

[33] Maier AD, Stenman A, Svahn F, Mirian C, Bartek J, Juhler M (2021). TERT promoter mutations in primary and secondary WHO grade III meningioma. Brain Pathol.

[34] Williams EA, Santagata S, Wakimoto H, Shankar GM, Barker FG, Sharaf R (2020). Distinct genomic subclasses of high-grade/progressive meningiomas: NF2-associated, NF2- exclusive, and NF2-agnostic. Acta Neuropathol Commun.

[35] Barresi V, Simbolo M, Fioravanzo A, Piredda ML, Caffo M, Ghimenton C (2021). Molecular profiling of 22 primary atypical meningiomas shows the prognostic significance of 18q heterozygous loss and CDKN2A/B homozygous deletion on recurrence-free survival. Cancers (Basel).

[36] Rutland JW, Gill CM, Loewenstern J, Arib H, Pain M, Umphlett M (2021). NF2 mutation status and tumor mutational burden correlate with immune cell infiltration in meningiomas. Cancer Immunol Immunother.

[37] Gill CM, Loewenstern J, Rutland JW, Arib H, Pain M, Umphlett M (2021). SWI/SNF chromatin remodeling complex alterations in meningioma. J Cancer Res Clin Oncol.

[38] Olar A, Wani KM, Wilson CD, Zadeh G, DeMonte F, Jones DTW (2017). Global epigenetic profiling identifies methylation subgroups associated with recurrence-free survival in meningioma. Acta Neuropathol.

[39] Sahm F, Schrimpf D, Stichel D, Jones DTW, Hielscher T, Schefzyk S (2017). DNA methylationbased classification and grading system for meningioma: A multicentre, retrospective analysis. Lancet Oncol.

[40] Nazem AA, Ruzevick J, Ferreira MJ. (2020). Advances in meningioma genomics, proteomics, and epigenetics: Insights into biomarker identification and targeted therapies. Oncotarget.

[41] Graillon T, Romano D, Defilles C, Saveanu A, Mohamed A, Figarella-Branger D (2017). Octreotide therapy in meningiomas: In vitro study, clinical correlation, and literature review. J Neurosurg.

[42] Graillon T, Defilles C, Mohamed A, Lisbonis C, Germanetti AL, Chinot O (2015). Combined treatment by octreotide and everolimus: Octreotide enhances inhibitory effect of everolimus in aggressive meningiomas. J Neurooncol.

[43] Graillon T, Sanson M, Campello C, Idbaih A, Peyre M, Peyrière H (2020). Everolimus and octreotide for patients with recurrent meningioma: Results from the Phase II CEVOREM Trial. Clin Cancer Res.

[44] Cardona AF, Ruiz-Patiño A, Zatarain-Barrón ZL, Hakim F, Jiménez E, Mejía JA (2019). Systemic management of malignant meningiomas: A comparative survival and molecular marker analysis between Octreotide in combination with Everolimus and Sunitinib. PLoS ONE.

[45] Hilton DA, Shivane A, Kirk L, Bassiri K, Enki DG, Hanemann CO. (2016). Activation of multiple growth factor signalling pathways is frequent in meningiomas. Neuropathology.

[46] Kaley TJ, Wen P, Schiff D, Ligon K, Haidar S, Karimi S (2015). Phase II trial of sunitinib for recurrent and progressive atypical and anaplastic meningioma. Neuro Oncol.

[47] Scerrati A, Mongardi L, Visani J, Lofrese G, Cavallo MA, Fiorentino A (2020). The controversial role of Bevacizumab in the treatment of patients with intracranial meningioma: A comprehensive literature review. Expert Rev Anticancer Ther.

[48] Franke AJ, Skelton WP, Woody LE, Bregy A, Shah AH, Vakharia K (2018). Role of bevacizumab for treatment-refractory meningiomas: A systematic analysis and literature review. Surg Neurol Int.

[49] Unterberger A, Nguyen T, Duong C, Kondajji A, Kulinich D, Yang I. (2021). Meta-analysis of adjuvant radiotherapy for intracranial atypical and malignant meningiomas. J Neurooncol.

[50] Goldbrunner R, Minniti G, Preusser M, Jenkinson MD, Sallabanda K, Houdart E (2016). EANO guidelines for the diagnosis and treatment of meningiomas. Lancet Oncol.

[51] Stögbauer L, Stummer W, Senner V, Brokinkel B. (2020). Telomerase activity, TERT expression, hTERT promoter alterations, and alternative lengthening of the telomeres (ALT) in meningiomas - a systematic review. Neurosurg Rev.

